# Stable Gastric Pentadecapeptide BPC 157 May Recover Brain–Gut Axis and Gut–Brain Axis Function

**DOI:** 10.3390/ph16050676

**Published:** 2023-04-30

**Authors:** Predrag Sikiric, Slaven Gojkovic, Ivan Krezic, Ivan Maria Smoday, Luka Kalogjera, Helena Zizek, Katarina Oroz, Hrvoje Vranes, Vlasta Vukovic, May Labidi, Sanja Strbe, Lidija Baketic Oreskovic, Marko Sever, Marijan Tepes, Mario Knezevic, Ivan Barisic, Vladimir Blagaic, Josipa Vlainic, Ivan Dobric, Mario Staresinic, Anita Skrtic, Ivana Jurjevic, Alenka Boban Blagaic, Sven Seiwerth

**Affiliations:** 1 Department of Pharmacology, School of Medicine, University of Zagreb, 10000 Zagreb, Croatialidijabaketicoreskovic@gmail.com (L.B.O.);; 2 Department of Surgery, School of Medicine, University of Zagreb, 10000 Zagreb, Croatia; 3 Department of Clinical Medicine, Faculty of Dental Medicine and Health, University of Osijek, 31000 Osijek, Croatia; 4 Department of Obstetrics and Gynecology, Clinical Hospital Sveti Duh, 10000 Zagreb, Croatia; 5 Laboratory for Advanced Genomics, Division of Molecular Medicine, lnstitute Ruder Boskovic, 10000 Zagreb, Croatia; 6 Department of Pathology, School of Medicine, University of Zagreb, 10000 Zagreb, Croatia

**Keywords:** gastric pentadecapeptide BPC 157, brain–gut axis, gut–brain axis, occlusion syndrome, occlusion-like syndrome, heart failure, encephalopathy, muscle

## Abstract

Conceptually, a wide beneficial effect, both peripherally and centrally, might have been essential for the harmony of brain–gut and gut–brain axes’ function. Seen from the original viewpoint of the gut peptides’ significance and brain relation, the favorable stable gastric pentadecapeptide BPC 157 evidence in the brain–gut and gut–brain axes’ function might have been presented as a particular interconnected network. These were the behavioral findings (interaction with main systems, anxiolytic, anticonvulsive, antidepressant effect, counteracted catalepsy, and positive and negative schizophrenia symptoms models). Muscle healing and function recovery appeared as the therapeutic effects of BPC 157 on the various muscle disabilities of a multitude of causes, both peripheral and central. Heart failure was counteracted (including arrhythmias and thrombosis), and smooth muscle function recovered. These existed as a multimodal muscle axis impact on muscle function and healing as a function of the brain–gut axis and gut–brain axis as whole. Finally, encephalopathies, acting simultaneously in both the periphery and central nervous system, BPC 157 counteracted stomach and liver lesions and various encephalopathies in NSAIDs and insulin rats. BPC 157 therapy by rapidly activated collateral pathways counteracted the vascular and multiorgan failure concomitant to major vessel occlusion and, similar to noxious procedures, reversed initiated multicausal noxious circuit of the occlusion/occlusion-like syndrome. Severe intracranial (superior sagittal sinus) hypertension, portal and caval hypertensions, and aortal hypotension were attenuated/eliminated. Counteracted were the severe lesions in the brain, lungs, liver, kidney, and gastrointestinal tract. In particular, progressing thrombosis, both peripherally and centrally, and heart arrhythmias and infarction that would consistently occur were fully counteracted and/or almost annihilated. To conclude, we suggest further BPC 157 therapy applications.

## 1. Introduction

The cytoprotective stable gastric pentadecapeptide BPC 157, by its effects, might combine the brain–gut and gut–brain axes’ function [1,2,3,4,5,6] in a particular way using its particular capabilities: cytoprotection [7] as a particular vascular effect [1], wound healing [8], and neuroprotection [9]. These were implemented in the most recently reviewed particular effects of the pentadecapeptide BPC 157 on the prompt activation of the vascular collaterals and counteraction of the occlusion/occlusion-like syndromes in rats with permanent major vessel occlusions and similar procedures severely disabling endothelium function, both peripherally and centrally, causing muscle disturbances in both striated and smooth muscles, disabling heart failure recovery as a whole [1,2,6,7]. Thus, these most recent findings [1,2,6,7] may readdress the particular issue of the brain–gut axis and gut–brain axis, using the original conceptual marks long ago proposed, starting with the gut peptides [10,11,12].

We emphasize the stable gastric pentadecapeptide BPC 157 as a capable treatment [7,8,9]. Peptide native and stable in the human gastric juice in all of the studies to date, consistent with the cytoprotection’s stomach background toward pleiotropic beneficial effects (i.e., stability in gastric juice more than 24 h ascertains easy applicability, even via the per-oral route), had demonstrated encouraging healing effects for various injury types in a variety of models, numerous organ systems, and different species. These might have been the particular effects on muscle function and vascular (activation of the collaterals) and nerve function, both peripherally and centrally [1,2,6]. This network might have been essential for BPC 157 therapy, with counteracted brain injuries [5,9], and the preserved muscle function might have been seen as a translation, consistently occurring as a well-functioning cytoprotection loop (i.e., brain–periphery) [1,2,7,8,9].

On the other hand, the significance of the stable gastric pentadecapeptide BPC 157 up for review for the brain–gut axis function [5,9] should deal with the brain–gut axis complex perception as an intriguing point at the time, providing more than 4900 studies for “brain-gut axis“ in Pubmed. To resolve this intriguing issue, it seems to us that both BPC 157 utility and brain–gut concepts in general should follow the initial definitions within the gut peptides [10] as the most valuable and indicative definition, as the “brain–gut axis“ maxim had originated within the gut peptides activities [10]. In this, the contemporaneous concept of Robert’s and Szabo’s cytoprotection theory [13,14,15,16,17,18,19,20,21] had also intended to describe the potential utility of gut peptides. It was a concept born in the stomach, holding direct epithelial cell protection; direct endothelium cell protection; and maxim endothelial maintenance to epithelial maintenance [13,16,19]. Illustrating the crucial events in the 1980s, the increased interest in the effects of the nervous system on the gut [10] was ascribed to the discovery of somatostatin in gastrointestinal and pancreatic D-cells in 1975 and, therefore, studies of the brain–gut relations (the brain–gut axis). In parallel, somatostatin was thought to be an innate cytoprotection mediator to translate the original stomach epithelial protection (cytoprotection) to the pleiotropic beneficial effect as protection of the other epithelia as well (i.e., liver) (organoprotection) [16]. In addition, the other peptides were isolated and sequenced from both the brain and the digestive tract (i.e., substance P [22,23], neurotensin [24,25], cholecystokinin (CCK) [26], vasoactive intestinal polypeptide (VIP) [27], and motilin [28]) were first isolated in the digestive tract and then found in the brain by immunoassay. The isolation of bombesin follows the amphibians’ skin, the brain [29], and the digestive tract [30]. Immunoassay revealed isolated brain peptides somatostatin [31], enkephalin [32], and the thyrotropin-releasing hormone (TRH) [33] in the digestive tract.

However, the needed reversal of the brain–gut axis function failure remained a challenge for the wide applicability of the original postulates of gut peptides’ brain–gut axis [10,11,12]. Likewise, Robert’s and Szabo’s cytoprotection concept, defined with the prostaglandin system [13,16,19], might also be questioned. As a whole, to demonstrate the needed reversal of the brain–gut axis function failure, the best approach may be with BPC 157 therapy [1,2,7,8,9]. Note, BPC 157 as a novel cytoprotection mediator, both native and stable in human gastric juice, might have an easy application [1,2,7,8]. Consequently, it might fully incline to the innate cytoprotection function, the continuous maintenance of the stomach and gastrointestinal mucosa integrity, and the pleiotropic beneficial effect [1,2,7,8,9]. Note, prostaglandin system function may be recovered by BPC 157, as BPC 157 therapy may antagonize all damaging effects of non-steroidal anti-inflammatory drugs (NSAIDs) [34]. In particular, BPC 157 therapy antagonized the combined NSAIDs-induced disturbances, both centrally and peripherally. Centrally counteracted were the various encephalopathies [35,36,37,38,39,40]. Likewise, the gastrointestinal and liver lesions along with the bleeding disorders were peripherally counteracted [35,36,37,38,39,40,41,42,43,44]. Accordingly, BPC 157 functions as an acting membrane stabilizer (leaky gut syndrome annihilated) [41] and a free radical scavenger [41,45,46,47,48,49,50]. This might occur particularly in vascular studies [51,52,53,54,55,56,57,58]. In addition, BPC 157 might interact with many molecular pathways [59,60,61,62,63,64,65,66,67,68]. Especially, it might interact with the nitric oxide (NO) system as a whole [69]. It might modulate its inhibition, overstimulation, and immobilization [69]; is likely to ascertain the NO system’s functioning (NO release of its own [60,69,70,71]; and the VEGFR2-Akt-eNOS signaling pathway might have been activated without the need for other known ligands or shear stresses, controlling vasomotor tone and the activation of the Src-Caveolin-1-eNOS pathway [59,60]). This follows BPC 157 as a novel cytoprotection mediator, is very safe, and has no side effects in clinical trials (i.e., used in ulcerative colitis), with a lethal dose (LD1) not reached in toxicology studies [1,2,3,7,8,9,72]. Moreover, it might have been a native peptide therapy with a high wound-healing capacity, with the simultaneous healing of different tissues (always used without any carrier addition) [8,73]. Most importantly, parenteral, intragastric, per-oral (in drinking water), and topical (i.e., cream, solution, eye drops) routes of administration suggest suited therapy applications [1,2,3,7,8,9,72,73].

At the general level, the organization in the brain–gut axis, as well as in the gut–brain axis, might have been quite complex. There is a commonly acknowledged large pathology involvement in the central nervous system, neuroendocrine system, neuroimmune systems, hypothalamic–pituitary–adrenal axis (HPA axis), sympathetic and parasympathetic arms of the autonomic nervous system, enteric nervous system, vagus nerve, and the gut microbiota [74,75,76,77,78]. On the other hand, at the practical level, the multitude of players that might have been involved axiomatically in the complicated and intertwined mechanisms include the ambiguous bidirectional directions of the change between the brain and the gut, either the brain–gut axis, the gut–brain axis, or both. Furthermore, in such undefined circumstances, the different mechanisms between animals and humans, animal strains, and possibly even individuals may be a further obstacle, as multiple mechanisms could also act in parallel [74,75,76,77,78].

Thus, at the present time, to form both theoretical and practical viewpoints suitable for the explication and implementation of the significance of the brain–gut axis or the gut–brain axis, as the activities of the brain–gut-axis and the gut–brain axis co-exist in particular, would require a particular focus, considering their organization and the involvement of the pathology. Therefore, we should reconsider the original principle [10]. The given prime leading role of the brain works with the first original players, well numbered and defined by the available peptides present at that time (by immunoassay) in both the central nervous system and the digestive tract (i.e., substance P, neurotensin, CCK, VIP, motilin, somatostatin, encephalin, TRH, and bombesin) [10]. This assumes that the agent’s activity (central) might verify the brain–gut axis activity [10]. This implies that the brain–gut and the gut–brain axes were defined specifically by the range of the effects of the proposed peptide agents [10].

Given the verified therapeutic effects of the brain–gut axis and the gut–brain axis, despite the particular mechanism [5,9], the resolving key would be within the gut peptides. Thus, we believe that a more extensive range of beneficial effects in the peripheral studies (in particular, in cytoprotective studies) would mean a more extensive range of beneficial effects in central nervous system studies and vice versa. Furthermore, most importantly, the more extensive range of the beneficial effect is noted simultaneously in both the periphery and central nervous system. Therefore, as such, the more accurate definition of the brain–gut axis and/or gut–brain axis involvement might serve to fully define potential agent applicability.

In this, the conceptual relation between Robert and Szabo’s stomach/cytoprotection and Robert and Szabo’s organoprotection, the protection of other tissues [13,16] should be more practically translated to that which would achieve the brain in particular [79]. This concept emerges as BPC 157 as a novel cytoprotection mediator because of its particular cytoprotective capabilities [1,2,3,7,8,9] as a peptidergic agent, native and not degraded in the human gastric juice, its stability exceeding a 24 h timeframe. Therefore, its cytoprotective capabilities are effectively translated into pleiotropic beneficial effects both peripherally and centrally [1,2,3,7,8,9,72,73]. An additional novel point, which would combine peripheral and central effects, might have been the particular vascular effect for epithelial and endothelial protection [1,2,3,7,8,9]. The upgrading of the minor vessel occurred rapidly. Then, it may take over and compensate for the function of the disabled major vessel. Activated collateral pathways depending on the given injury (i.e., activated azygos vein direct blood flow delivery) may reestablish the reorganized blood flow [1,2]. This might arise from the original BPC 157 cytoprotective evidence (i.e., maintenance of the endothelium integrity) [1,2,3,7,8,9]. This appeared as an essential and particular rapid therapeutic effect. Consistent efficacy occurred against devastatingly present Virchow triad circumstances. The counteraction included the major vessel occlusion [51,52,53,54,67,68,80,81] and other similar noxious procedures known to disable severely endothelium function [56,57,82,83,84]. Severe vessel and multiorgan failure syndrome, occlusion/occlusion-like syndrome, severe intracranial (superior sagittal sinus) hypertension, portal and caval hypertensions, and aortal hypotension occurred and were all attenuated/eliminated by BPC 157 therapy. Consistent with BPC 157 therapy, the severe lesions in the brain, heart, lung, liver, kidney, and gastrointestinal tract were counteracted, and, in particular, progressing thrombosis, both peripherally and centrally, was almost annihilated, and the counteractions of heart failure, arrhythmia, and infarction consistently occurred [1]. Likewise, for these beneficial effects, the upgraded venous system might have been essential [51,52,53,54,56,57,67,68,80,81,82,83,84]. Of note, these BPC 157 pleiotropic beneficial effects might evidently implement the original meaning of Robert epithelium maintenance, Szabo endothelium maintenance, and the endothelium maintenance to epithelium maxim maintenance [13,14,15,16,17,18,19,20,21].

Conceptually, the as such consequent “direct cell protection” [13] axiomatically precludes any adverse effects consequent to the cytoprotective agent’s application (although the applications of the standard cytoprotective prototype agents and prostaglandins agents might result in considerable concomitant adverse effects) [85]. Thus, with BPC 157 therapy, epithelium/endothelium maintenance, and the endothelium maintenance to epithelium maintenance maxim [13,19] might have been the rapid basis for the rapid upgrading of the minor vessel [1]. This might have been the substituting function for the failed major vessel, reestablishing reorganized blood flow via recruited collaterals (azygos vein direct blood delivery) in either ischemia or reperfusion conditions [1,2,7]. The given therapeutic effects of BPC 157 therapy might have been illustrative for the initial heart’s infarct induction and re-infarction [57]. It occurred combined with the consistent anti-thrombosis [51,52,53,54,56,57,67,80,81,82,83,84]. Additionally, it occurred along with an anti-arrhythmic effect [51,52,53,54,56,57,67,80,81,82,83,84,86]. Additionally, therapy in the reperfusion after bilateral clamping of the common carotid arteries for a 20 min period [68] showed the effects of BPC 157 [57] in parallel with those that might occur in stroke in rats. The therapy counteracted both early and delayed neural hippocampal damage, achieving full functional recovery (Morris water maze test, inclined beam-walking test, lateral push test) [68], as assessed at 24 h and 72 h of the reperfusion.

Conceptually, such a wide beneficial effect, both peripherally and centrally, might have been essential from the viewpoint of the brain–gut and gut–brain axes’ function harmony [5,9]. However, as seen from the original viewpoint of the gut peptides’ significance and brain relation, the favorable evidence of BPC 157 in the brain–gut and gut–brain axes’ function might have been reviewed as a particular network of mutually interconnected beneficial activities. These were the behavioral findings (Section 2); muscle healing and function recovery findings (Section 3); various encephalopathies, occlusion/occlusion-like syndromes, and particular vascular collateral pathways activation findings (Section 4); and progressive thrombosis counteraction findings (Section 5).

## 2. Behavior

In general, the potential holding of the behavioral disturbances and therapeutic effects of the agents, such as BPC 157, with logistical assistance from the many essential systems (i.e., GABA, opioids, dopamine, serotonin, and NO system) [5,9] largely implemented in physiology and pathology, might have been an indicative link for possible significances and therapies. On the other hand, it might spark a fierce controversy between the value of animal models and their potential translations to the human condition [87]. This might have been the case with some particular modulatory effects of BPC 157 therapy. They might have been particularly presented and commonly applicable when these systems might have been disabled in either way [5]. The above-mentioned particular modulatory effects, encompassing the entire NO system [69], may be a particular illustration of a complex effect that regularly resolves activity. First, BPC 157-induced NO is released on its own [60,70,71], which may be resistant to N(G)-nitro-L-arginine methylester (L-NAME)-induced NO synthase (NOS) inhibition. Then, there is a counteraction of NO synthase (NOS) inhibition (i.e., L-NAME-hypertension and pro-thrombotic antagonized effects may be particular examples) [43,70]. Finally, NO over-stimulation was also antagonized (antagonization of L-arginine-hypotension and anti-thrombotic, pro-bleeding effects may provide indicative examples) [43,70]. Likewise, the counteraction of the isoprenaline myocardial infarction by BPC 157 therapy might include a NO effect [57]. Given the BPC 157–dopamine–NO system interaction (i.e., BPC 157 therapy might antagonize L-NAME-induced catalepsy) [88], such an interaction might support the hypothesis that BPC 157 therapy might exert two seemingly opposite effects. It might consistently antagonize the effect of the dopamine receptors’ blockades as well as antagonize the effect of the dopamine receptors’ agonization/over-stimulation [88,89,90,91,92]. With these caveats, the evidence concerning the particular beneficial effects of BPC 157 therapy (anxiolytic; anticonvulsive; antidepressant; catalepsy counteraction, which might have been used in positive and negative schizophrenia symptom models) within the brain–gut axis implementation (i.e., simultaneous effect, centrally and peripherally) will be presented.

A particular anxiolytic effect appeared with the antagonization of the most serious side effects of benzodiazepine therapy, the development of tolerance, and physical dependence, all of which were attenuated [91]. Illustratively, a later, acute administration of diazepam together with convulsant demonstrated a BPC 157-attenuated diazepam tolerance. The later administration of isoniazid evidenced the antagonization of a postponed physical dependence/withdrawal effect [91]. Therefore, it might have been a particular anxiolytic effect (i.e., not burying and no more shocks (shock probe/burying) and a greater number of crossing and exploratory rearing behaviors in a dark areas (light/dark test)) [93]. In addition, this effect might affect general anesthesia as well [94] (i.e., BPC 157 antagonized thiopental-induced general anesthesia (parallel shift of the dose–response curve to the right) [94]). In this, illustrative of the antagonization of ethanol disorders, BPC 157 was suggested to act as an antagonist of ethanol [85,95], counteracting both acute and chronic alcohol intoxications [96,97]. Furthermore, such an antagonization might have been a particular point given to anesthetic barbiturates, such as thiopental, and the counteraction of their activities as both agonists at GABAA receptors and enhancers of receptor responses to GABA [98]. Additionally, BPC 157 exhibited an intrinsic anti-convulsive activity and counteracted convulsions induced by picrotoxin, isoniazid, and bicuculline [91,99], known as the non-competitive blocker of GABA-receptor chloride channels, the inhibitor of glutamic acid decarboxylase and GABA-synthesis, or the GABAA antagonist [100,101,102]. Thus, BPC 157 might act to favor the natural homeostasis of the GABA receptor complex as well as to enhance GABAergic transmission. In addition, there was an additional anxiolytic effect in the counteraction of negative schizophrenic symptoms in rats dosed with ketamine [103]. Importantly, these effects were shown to be related to the NO system effects as well [94,103].

Finally, the full complexity of the brain–gut axis function might have been illustrated with the application of alcohol and the extent of the antagonization disturbances. The counteraction included behavioral disturbances in acute and chronic alcohol intoxications [96,97]. Additionally, those counteracted were hypothermia, mucosal and endothelial lesions, liver injuries, and portal hypertension [70,82,96,97,104,105,106]. Moreover, an additional illustration of both the extensive lesions and BPC 157 therapy potentials appeared in absolute alcohol intragastric administration, producing severe occlusion/occlusion-like syndrome. BPC 157 therapy, as a part of the counteraction of the severe vessel and multiorgan failure syndrome with the activation of the collateral rescuing pathway of the azygos vein (direct blood flow delivery), counteracted brain, lung, liver, kidney, and gastrointestinal lesions; progressive thrombosis, both peripherally and centrally; and, in particular, heart failure, arrhythmia, and infarction [82]. As an instant effect, those counteracted were intracranial (superior sagittal sinus) hypertension, portal and caval hypertensions, and aortal hypotension.

The development of serotonin syndrome belongs to the most serious side effects of antidepressant therapies. BPC 157 therapy might fully counteract serotonin syndrome [107]. Namely, the irreversible monoamine oxidase (MAO) inhibition (i.e., pargyline) and subsequent serotonin substrate (L-tryptophan as a serotonin precursor) induced fore paw treading, hind limbs abduction, wet dog shake, and hypothermia followed by hyperthermia in rats, which commonly occur in serotonin syndrome [107]. Both temperature and behavioral changes in all these experiments were counteracted by gastric pentadecapeptide BPC 157. This effect may have been a particular effect, as BPC 157 counteracted each part of the serotonin syndrome presentations. First, BPC 157 therapy counteracted serotonin syndrome initiation (i.e., counteracted pargyline effect) [107]. Then, in particular, BPC 157 counteracted the full serotonin syndrome crisis (attenuated the adverse effect of the subsequent L-tryptophan application) [107]. Thus, BPC 157 had a special effect. It was not acting as a serotonin substrate, nor was it able to induce serotonin syndrome (as did L-tryptophan). Therefore, antagonizing all aspects of serotonin syndrome might substantiate the significance of BPC 157 on its own [107]. In support, alpha-[14C]methyl-L-tryptophan autoradiographic measurements confirmed that the effects of BPC 157 were distinguished [108] from the effects of any other serotonergic drugs [109,110,111,112]. The peripheral application of BPC 157 as a gut peptide had region-specific influences on brain serotonin synthesis in rats in both acute and chronic treatments [108]. As always, increased serotonin release was particularly related to the innate effect on the substantia nigra structure [108]. As additional support, BPC 157 therapy reduced antidepressant arrhythmias and severe cardiotoxicities, exhibited considerable anti-arrhythmic potential against various arrhythmias, and opposed heart failure disturbances [2,51,52,53,54,56,57,67,80,81,82,83,84,86,113,114]. Finally, in classic antidepressant assays, BPC 157 therapy (Porsolt’s test, chronic stress, reduced duration of immobility) overwhelmed the effect of imipramine [115].

Finally, the evidence that the gut produces 95% of the serotonin in the human body [65] might illustrate the full complexities of the serotonin system’s brain–gut axis function and the gut–brain axis, likely also related to the effects of BPC 157. BPC 157 in the gastrointestinal tract reduced the release of enteric serotonin and suppressed intestinal motility [65]. These were along with the increase in the survival rate of cultured enteric neurons and the proliferation of cultured enteric glial cells. These were thought to be indicative of the improved healing of damaged enteric nervous and mucosal structures; cytoprotection functioning, both peripherally and centrally; and the simultaneous functioning of both the brain–gut axis and the gut–brain axis [65].

For the interaction of BPC 157 and dopamine [5,9], common clarification and recovery of the dopamine agents’ adverse effects as novel common points might have been particularly interesting. Namely, they are in a class of psychotropic medications, primarily used either to manage psychosis and bipolar disorder (along with mood stabilizers) [116,117] or as central nervous stimulants in the treatment of attention deficit disorder, narcolepsy, and obesity [118,119].

An ideal agent would be able to affect the dopamine system’s function and cause a therapeutic effect only at the site of a pathologic brain–gut axis and/or gut–brain axis function. In this, BPC 157 may have a complex therapeutic effect. BPC 157 largely interacts with the dopamine system [5,9] since BPC 157 had counteracted various behavioral disturbances, tremors, akinesia, and catalepsy as well as stereotypies that appear within the dopamine system’s disability, its function either reduced or over-amplified [88,89,90,92].

First, the particular points were the destruction of the brain’s dopamine areas, vesicle depletion, and the blockade of the dopamine receptors. BPC 157 therapy had counteracted disturbances caused by the application of the parkinsongenic neurotoxin 1-methyl-4-phenyl-1,2,3,6-tetrahydrophyridine (MPTP) and vesicle depletion by reserpine application [120]. Likewise, BPC 157 counteracted the effects of dopamine receptor blockades that would appear in applications of haloperidol, fluphenazine, clozapine, and sulpiride [46,70,88,89,121,122,123,124], both peripherally and centrally. Additionally, it might interfere with the interactions of the opioid–dopamine system, antagonizing the haloperidol potentiation of morphine analgesia and, also, morphine analgesia alone [125].

Second, to emphasize the opposite over-activity disturbances and the complexities of the likely important modulatory role of BPC 157 in dopamine–BPC 157 interactions, we should accentuate that BPC 157 counteracted disturbances (i.e., stereotypies) in acute and chronic amphetamine applications (i.e., tolerance and reverse tolerance) [90,92]. Additionally, BPC 157 therapy had antagonized disturbances that were characteristic in the course of the amphetamine, methamphetamine, apomorphine, and dopamine (over)-stimulation in the suited models of the positive-like schizophrenia symptoms [88]. Furthermore, these effects were extended to ketamine as a noncompetitive NMDA antagonist [126,127], and consistent counteractions of negative-like symptoms of schizophrenia occurred in particular relation to the NO system’s functioning [103]. BPC 157 had a full therapeutic effect with the counteraction of ketamine–cognition dysfunction, social withdrawal, and anhedonia, and it had exerted additional anxiolytic effects [103]. Note, all the used models were commonly used (i.e., novel object recognition test for cognitive dysfunction [128,129,130], open arena and social interaction for social withdrawal [131,132], sucrose test for anhedonia [133,134], and open field test for anxiogenic effects [134,135,136,137]). Therefore, the reported full therapeutic effects may have been important [103]. In comparison, the effects of the NO agents were more limited. The therapeutic effects of L-NAME (antagonization, social withdrawal) and the therapeutic effects of L-arginine (antagonization, cognitive dysfunction, anhedonia) had both included: worsening cognitive dysfunction, anhedonia, and anxiogenic effects (L-NAME) and social withdrawal and anxiogenic effects (L-arginine) [103]. Indicatively, BPC 157 counteracted these aggravations [103]. Note, acute ketamine administration had a particular point, as it was associated with schizophrenia-like or psychotomimetic symptoms with large effect sizes and an increase in positive and negative symptoms [126]. Thus, the ketamine model made the particular model of schizophrenia. Therefore, the extraordinary complexities of mental disorder extrapolation from animal models in general [87] may less dispute the ketamine model’s result [103].

Furthermore, the involvement of the dopamine system in the full complexities of the brain–gut axis function and the gut–brain axis might have been illustrated with Szabo dopamine anti-parkinsonian agents as a therapy for peptic ulcers [19,138] and, therefore, dopamine agonists in peptic ulcer therapy to counteract duodenal ulcer recidives [139]. At a particular point, both schizophrenia and the diminished risk for duodenal and gastric ulcers were claimed together [140]. These also illustrated the evidence that dopamine antagonists alone might produce gastric ulcers [141,142], which dopamine agonists or BPC 157 therapies might have antagonized, but not standard anti-ulcer agents [70,121,123]. Likewise, this might also be with the counteraction of the reserpine- and MPTP-induced gastric ulcers, the application of combined reserpine and dopamine antagonists, the depletion of dopamine vesicles, and the blockade of receptors [120,121]. Consistently, BPC 157 had also counteracted, consequently, lower esophageal and pyloric sphincter dysfunctions, induced by dopamine antagonists [46]. Similarly, BPC 157 therapy might antagonize dopamine antagonist (neuroleptics, typical and atypical, and prokinetics)-induced prolonged QTc intervals that occurred as a central effect [122].

There might also be a further indicative key focus to indicate the particular potential of BPC 157 as an acting modulator. This might have been the counteracting of its behavioral supersensitivity to the amphetamine-stimulating effect. In principle, the increased amphetamine-climbing behavior following a dopamine antagonist haloperidol application was confirmation of the striatal dopamine receptor up-regulation and the subsequent development of amphetamine supersensitivity [143,144,145]. Illustratively, in mice pretreated with haloperidol, and, subsequently, challenged with amphetamine [90], using the described procedure [143], this pentadecapeptide antagonized increased climbing behavior [90]. This might verify the fully avoided striatal dopamine receptors’ up-regulation and supersensitivity [90]. Thus, the haloperidol–amphetamine interaction fully antagonized by BPC 157 may suggest a modulatory effect that is evidently distinctive from any effect that might potentiate haloperidol–amphetamine interactions, dopamine receptor up-regulations, and increased sensitivities to amphetamines (i.e., neither additional dopamine antagonist nor dopamine agonistic activity, either direct or indirect) [5,90]. Thus, such an overwhelming modulatory effect on the dopamine system might substitute, to some extent, the function of the otherwise prominently insufficient dopamine system. This means avoiding the subsequent dopamine receptor up-regulation and counteracting raised amphetamine supersensitivity disturbances [5,9]. Consequently, this explains that it both prevents and reverses the consequent disturbances (i.e., stereotypic behavior) in confrontation with the increased amphetamine-induced dopamine release and synthesis [88,90,92]. A similar confirmation of this particular modulatory role is that it could markedly attenuate the consequence of a dopamine receptor blockade through haloperidol and other dopamine receptor antagonists [88,89]. Moreover, the BPC 157’s counteractive effects on catalepsy and positive-like or negative-like schizophrenia symptoms models was substantiated in relation to NO agents as a final therapy outcome: BPC 157 ˃ L-arginine ˃ L-NAME. This conclusion was further substantiated with the NO agents’ triple application (regularly used in our BPC 157 studies), thereby covering all the NO system’s functioning, blockades (L-NAME), over-stimulations (L-arginine), and immobilizations (L-NAME+L-arginine) [88,103].

Furthermore, BPC 157’s special vascular effect (activation of the collateral pathway) [1] might have been an additional clarification of the involvement of the full complexities of the brain–gut axis function together with the gut–brain axis function. As indicated before for the counteraction of absolute-alcohol-intragastric-administration-induced occlusion/occlusion-like syndrome with the activation of the collateral pathway (azygos vein direct blood flow delivery) [82], BPC 157 therapy may, in the same particular way, counteract the occlusion/occlusion-like syndrome induced with lithium, which is known to interact with dopamine and serotonin systems and is a prototypic agent in bipolar disorder therapy [56]. Therefore, the counteracted lithium-induced multiorgan failure, both peripherally and centrally, the particular lithium-induced central and peripheral vascular failure, and the recovery of the lithium-disabled dopamine and serotonin systems may be seen as equal recoveries of vascular failures by BPC 157 therapy [56]. Such particular vascular recoveries may interact with NO-specific molecular pathways [59,60] (i.e., BPC 157 regulates vasomotor tone and the activation of the Src-Caveolin-1-endothelial NOS pathway [60]).

Additionally, these described vascular recoveries [56,82] may be essential in strongly supporting the comparable therapeutic effects of BPC 157 on stroke in rats [68]. Therapy was initiated in the reperfusion after the bilateral clamping of the common carotid arteries for a 20 min period [68] and provided a large beneficial effect on brain lesions (i.e., at 24 h and 72 h of the reperfusion, the therapy had counteracted both early and delayed neural hippocampal damage). An achieved fully functional recovery (Morris water maze test, inclined beam-walking test, lateral push test) [68] evidently ascertained the translation of the central beneficial effects to the preserved peripheral functions (i.e., muscle function). This may be regarded as a well-functioning cytoprotection loop (brain–periphery) that might consistently occur as an implementation of the full complexities of the brain–gut axis function in BPC 157 therapy. Similar evidence of BPC 157 therapy might have been beneficial to the concussive brain traumas and preserved righting reflexes in the concussed mice [146]. Likewise, tail function was restored with BPC 157 therapy in rats after spinal cord compressions and tail paralyses [147,148]. An instructive example might also be the counteracted effect of the cuprizone, a neurotoxin commonly used to produce multiple-sclerosis-like lesions in rats, attenuated brain lesions, and recovered muscle functions [149].

These data are summarized in Table 1.

## 3. Muscle

The BPC 157 muscle–brain perception [5,9] might depart from the original brain–gut axis’s perception explained with gut peptides and smooth muscles [10]. As a complex whole, the motile system of the gastrointestinal tract (i.e. having several organizational levels) and central nervous system effects (i.e. to centrally modulate the long pathway reflexes traveling in the main vagosympathetic trunks) [10] occurred in the original gut peptides’ perception of the brain–gut axis function. Thus, the gut peptide effect accordingly occurred as part of the brain–gut functioning system. However, such contention about the gastrointestinal–smooth muscle–brain relation [10] might have been insufficient since it lacks consideration of the effects on the striated muscle’s healing and function. Namely, there are several important points providing the peptide agent’s activity consistently considered for muscle healing and function to be also revealed in the general term of the realization of brain-gut axis and gut-brain axis functioning [5,9]. Evidently, any purposive movement requires the impulses to pass from the motor cortex via the spinal cord to the appropriate muscles. Likewise, the impulses passing through various parts of the brain, sending messages back to the motor cortex [5,9], evidence the movement pattern as a highly coordinated event.

On the other hand, in this more extensive way for the brain-gut axis and gut-brain functioning, just with the BPC 157 therapy, implementing cytoprotection (simultaneous healing of different tissues, i.e. brain and periphery), we envisaged a multimodal muscle axis function [2,6]. Commonly, these may be the general terms of the realization of the brain-gut axis functioning [2,6]. With the BPC 157 therapy the described improved purposive movement rationale (via the motor cortex -spinal cord—appropriate muscles and vice versa) might conceptualize in the brain–muscle axis and muscle–brain axis bidirectional functioning the healing and function recovery of the myotendinous junction (dissection) [50], the muscle lesion (transection, contusion, and corticosteroid application), and nerve (transection) [50,150,151,152,153,154]. With muscle weakness, these might perceive both prime (i.e. brain) and secondary (muscle weakness) injury, prime lesion→secundary lesion, given the muscle weakness as part of the prime (i.e. brain injury) disturbance that has to be also attenuated when muscle weakness is attenuated/reversed (prime lesion↔secondary lesion). Thereby, the multitude of relations might be illustrative. Along with counteracted muscle weakness counteracted was a vascular failure [51,52,53,54,56,57,67,80,81,82,83,84]. Likewise, along with counteracted muscle weakness, counteracted were stroke [68], traumatic brain injury [146], neurotoxin cuprizone–induced multiple sclerosis-like brain lesions in rats [149], and spinal cord compression lesions [147,148]. Accordingly counteracted were the severe electrolyte disturbances and brain lesions [56,155,156,157]. More specific therapy targets of muscle disability counteractions considered the counteraction of succinylcholine-induced neuromuscular junction blockades [158], the antagonization of local anesthesia (intraplantar, axillar, intra-thecal [159], and corneal [160]), and the counteraction of additional adverse effects of local anesthetics (i.e., tetracaine, oxybuprocaine (i.e., dry eye [160]), and lidocaine (arrhythmias, seizures) [159]). Those antagonized were catalepsy, akinesia, and tremors with neuroleptic dopamine blockades [88,89,90], NO system blockades [88], applications of the parkinsongenic neurotoxin MPTP, and vesicle depletion by the application of reserpine [120]. In alcohol intoxication [96,97] and serotonin syndrome [107], muscle disturbances were counteracted along with the antagonization of the whole syndrome. There was also the counteraction of arterial and venous thrombosis in vessel occlusion and similar noxious procedures [51,52,53,54,56,57,67,80,81,82,83,84]. Tumor-induced muscle cachexia (i.e., muscle degeneration, inflammation, and catabolism) was antagonized, and the survival rate was increased [45]. Illustratively, this significant mitigating action against cancer-cachexia-induced corrected deranged muscle proliferation, as well as myogenesis, counteracted an increase in pro-inflammatory and pro-cachectic cytokines (i.e., interleukin 6 (IL-6) and TNF-α) implicated in muscle metabolism relevant to cancer cachexia. Likewise, counteraction occurred with any changes in the expression of FoxO3a, p-AKT, p-mTOR, and P-GSK-3β [45]. Note, in counteracting leaky gut syndrome, BPC 157 had a role of a membrane stabilizer [41] and a free radical scavenger [41,45,46,47,48,49,50]. These occurred particularly in vascular studies [51,52,53,54,55,56,57,58] and might have had distinctive beneficial effects. Thus, the muscle disturbances induced, and along with the given prime lesion, consistently counteracted (prime lesion↔secondary lesion), might be the common point essentially combining the mentioned multitude of injurious events and pleiotropic therapeutic potential.

Evidently, BPC 157 therapy substantiated the consistently preserved and recovered muscle function in all these experiments. These were the preserved Morris water maze test, the inclined-beam-walking test, the lateral push test (stroke rats) [68], the righting reflex (concussed mice) test [146], the counteracted ataxia and impaired forelimb function (cuprizone-induced multiple sclerosis-like rats) test [149], and the test to regain tail function after tail paralysis (spinal cord compression) [147,148]. This may be the translation to preserve muscle function that might consistently occur as a well-functioning cytoprotection loop (i.e., brain–periphery) as a part of the realized brain–gut axis central–peripheral functioning on both sides (for review see, i.e., [1,2,6,7]). To this point, the equal counteraction of various brain and spinal cord lesions may be illustrative. Those counteracted were both early and delayed neural hippocampal damages (stroke rats) [68]; brain lesions (concussed mice) [146]; lesions in various brain areas, with the most prominent damages in the corpus callosum, laterodorsal thalamus, nucleus reunions, and anterior horn motor neurons (cuprizone-induced multiple sclerosis-like rats) [149]; and markedly attenuated spinal cord compression hematoma and lesions (spinal cord compression) [147,148].

Within the same dosage range, there was a similar counteraction of myocardial infarction and myocardial reinfarction, along with brain injury mitigation and the counteraction of severe vascular and multiorgan failure (activated azygos vein direct blood flow delivery) [51,52,53,54,56,57,67,80,81,82,83,84]. In the intoxicated rats overdosed with lithium, severe myocardial brain lesions and muscular weaknesses were timely correlated, and they were consistently counteracted by the therapy [56]. Thus, these combined findings illustrated that, with BPC 157 therapy, a well-functioning cytoprotection loop (brain–periphery) largely occurred [5,9]. On the contrary, the cytoprotection loop (brain–periphery) seemed to be not operative with the standard cytoprotective agents’ stroke therapy (i.e., calcium channel blocker, isradipine) [161], and standard cytoprotective therapy exhibited only an incomplete effect [161] (i.e., decreased infarct size without preservation of the muscle function during the acute post-occlusion period).

Thus, the heart failure was counteracted (including arrhythmias and thrombosis counteracted) [2,51,52,53,54,56,57,67,80,81,82,83,84,86,113,114] in addition to the BPC 157 therapy effect on the various muscle disabilities of a multitude of peripheral [6] and central [6] causes. There, the recovery of the smooth muscle function was also shown, and we claimed the multimodal muscle axis impact [6], and the muscle function and healing as a function of the brain-gut axis and gut-brain axis as a whole [6].

Many smooth muscles appeared as a particular target of BPC 157 therapy. The beneficial effect occurred on many sphincters during the conditions of sickness. The recovery of the sphincters’ distinctive functions (lower esophageal sphincter, pyloric sphincter [46,95,106,162,163,164,165,166,167,168], pupil [169,170], urinary sphincter [48,171,172]) suggests that BPC 157 therapy might exert a distinctive effect. This effect may be dependent on the given lesion and condition, the maintained normal function, or the recovery of the disturbed function. This may preserve normal functioning anti-reflux effects (increases lower esophageal sphincter pressure, decreases pyloric sphincter pressure [162]) or maintain normal pupil diameter [169] or normal leak point pressures [171]. Recovery of sphincter failure occurred against a variety of agents and procedures. These included absolute alcohol [95,106], NSAIDs and/or neuroleptics and/or NO agents [46,167], tube insertions into sphincters [162,163,164], acute pancreatitis (bile duct ligation) [164], creation of fistulas [58,166,172], and the particular creation of anastomoses [168] (the lower esophageal sphincter, pyloric sphincter). Atropine [169], NO agents, L-NAME and/or L-arginine [169], and glaucoma (episcleral veins cauterization) [170] induced pupil dysfunction. Transabdominal urethrolysis, prolonged vaginal dilatation [171], and cyclophosphamide caused urinary sphincter dysfunction [48]. Those counteracted were different (even opposite) dysfunctions, i.e., NOS blockades, NOS over-activities, mioses, and mydriases [46,106,169]. Thus, with BPC 157 therapy, the maintenance or recovery of the function of the sphincter is operative against different peripheral and/or central insults (i.e., esophagitis [46,58,95,106,162,163,164,166,167], glaucoma [169,170], stress urinary incontinence [171], and cytostatic-induced bladder dysfunction [48]).

Generally, sphincter recovery occurred along with the recovery and healing of the prime causative lesions (peripheral and/or central) [46,48,106,162,163,164,165,166,167,168,169,170]. Illustrative findings occurred in the rats with intragastric absolute alcohol challenges [82,106]. The dysfunctions in the lower esophageal sphincters and pyloric sphincters were reversed, along with the counteraction of brain injuries, internal organ lesions, intracranial hypertension (superior sagittal sinus), portal and caval hypertensions, aortal hypotension elimination/counteraction, and vascular recovery (i.e., azygos vein direct blood delivery) [82,106]. In addition, there was also the recovery of the somatosensory neurons [173]. In the haloperidol rats with disabled sphincters, BPC 157 therapy counteracted oxidative stresses in the lower esophageal sphincters, pyloric sphincters, and brain [46]. In the eye, BPC 157 therapy annihilated both mydriasis and the counteraction of the glaucoma presentation in parallel [170]. Thus, the rats with cauterized episcleral veins had normal pupil diameters, well-preserved ganglion cells and optic nerve presentations, normal fundus presentations, normal retinal and choroidal blood vessel presentations, and normal optic nerve presentations as therapy outcomes [170].

In addition to these findings, with BPC 157 therapy both per-orally and parenterally, there was intestine recovery after massive intestinal resection, as well-controlled adaptive processes adequately affecting the entire intestinal wall (villus height, crypt depth, and muscle thickness (inner (circular) muscular layer) all accordingly increased [40,174,175], achieving full intestinal anastomosis healing [40,174,175] in particular. Constant weight gain above preoperative values occurred immediately with BPC 157 therapy [40,174,175]. In addition, BPC 157 increased the survival rate of the cultured enteric neurons, increased the proliferation of cultured enteric glial cells as part of the advanced cytoprotective effect, and suppressed motility in the gastrointestinal preparations, including human intestinal strips and rat strips [65]. Therefore, the counteracted brain lesions that otherwise might occur regularly after massive bowel resections [40,174] may suggest full the recovery of the brain–gut and gut–brain axes’ function. The role of BPC 157 was combined with the reduced release of enteric serotonin [65] along with the key role of enteric serotonin in physiological actions, the pathogenesis of intestinal inflammation, and had a prominent role in inflammatory bowel disease [176,177,178]. Additionally, significant modulation of the regional synthesis of serotonin in different areas of the rat brain might occur [108,179,180]. In particular, the increased release of serotonin, acutely and chronically, occurred in the substantia nigra [108].

The effect on smooth muscle might also be through the NO system, given that high concentrations of BPC 157 increased vasorelaxation in the aorta without endothelium [60], while BPC 157 modulated the vasomotor tone of an isolated aorta in a concentration- and nitric oxide-dependent manner [60] and in an induced NO generation manner, likely by activating the Src-Cav-1-eNOS pathway [60]. These might also relate to direct NO stimulation [43,69,70,71] as a part of its modulatory effect, the counteraction of the NOS blockade (i.e., L-NAME-hypertension and pro-thrombotic effect), and the counteraction of NOS over-stimulation (i.e., L-arginine-hypertension and anti-thrombotic effect) [43,70]. The VEGFR2-Akt-eNOS signaling pathway might have been directly activated without other known ligands or shear stresses controlling vasomotor tones and the activation of the Src-Caveolin-1-eNOS pathway [59,60].

Concluding, BPC 157 muscle/brain perception [5,9] might provide the full involvement of the muscle, striated muscle—heart muscle—smooth muscle. A consistent network of evidence for a multimodal muscle axis function might fully substantiate the background even beyond the original brain-gut axis perception explained with gut peptides [10]. 

These data are summarized in Table 2.

## 4. Brain Injury Concomitant Pathology

The more and more extensive range of beneficial effects should be simultaneously present in both the periphery and central nervous system to give rise to the brain–gut and gut–brain axes’ function as a verified therapeutic effect. Despite the particular mechanism, the resolving key highlighted the concept of cytoprotection. Illustratively, as defined within prostaglandin in the stomach and other tissues [13], with the general prostaglandin system significance, these arguments were provided with the counteraction of the noxious course following NSAIDs [34] by BPC 157 therapy. These were non-specific NSAIDs [36,37,38,40,41,42,43,44] as well as specific NSAIDs [39]. There were simultaneous counteractions of both central and peripheral injuries [36,37,38,39,40].

To analyze the obtained large range of the therapeutic effects of BPC 157, several particular points in NSAIDs-induced lesions (paracetamol, diclofenac, ibuprofen, and celecoxib) should be highlighted [36,37,38,39,40,41,42,43,44] with the application of overdosed NSAID regimens. For example, centrally, the particular aspect of the sudden onset of encephalopathy with severe seizures (paracetamol) or prolonged sedation and/or unconsciousness (diclofenac, ibuprofen) might have been indicative; the worst lesions were in the cerebellum and more in the white matter (paracetamol) or in the cerebral cortex and cerebellar nuclei in the Purkinje cells (diclofenac, ibuprofen, and celecoxib). Peripherally, huge liver lesions (paracetamol) or gastrointestinal and liver lesions (diclofenac, ibuprofen (hepatomegaly), and celecoxib) occurred. Therefore, it might have been that the recovery of the essential cytoprotective prostaglandin system, which was supposed to be inhibited with NSAIDs, occurred as the key to reestablishing brain–gut axis function. The effective, therapeutic μg-ng range of BPC 157, simultaneously occurring peripherally and centrally, might have been illustrative [36,37,38,39,40,41,42,43,44]. The wide applicability to the effective regimen might also be illustrative. The regimen given was intraperitoneally, intragastrically, or per-orally in drinking water. It might have been given in the early stage (i.e., BPC 157 intraperitoneal or intragastric application immediately after paracetamol, prophylactically, intraperitoneally immediately after diclofenac). Alternatively, the regimen was given in the advanced stage (i.e., BPC 157 at 3 h after paracetamol, therapeutically, or late after diclofenac in drinking water) [36,37,38]. The beneficial effect of the therapy occurred against all NSAIDs and involved the counteraction of all lesions. The counteraction of the brain lesion occurred along with the counteraction of the liver and gastrointestinal lesions. Illustratively, in paracetamol, rats regularly had very durable convulsions (lasting from 25 min to 5 h post-application time). BPC 157 demonstrated that convulsions did not appear (prophylactic application) [36]. Likewise, applied 3 h after paracetamol, when BPC 157 was confronted with advanced convulsions, the convulsions rapidly disappeared (therapeutic effect within 25 min) [36]. In addition, BPC 157 counteracted aspirin bleeding and consequent thrombocytopenia, maintaining thrombocyte function [42,43,44]. Finally, there was also a full counteraction of leaky gut syndrome [41].

Indicatively, these lesions and their worsening effects were related to the NO system. In short bowel rats (i.e. massive intestinal resection-, massive intestinal resection-plus-diclofenac-, and massive intestinal resection-plus-diclofenac-plus-L-NAME-treated (cyclooxygenase (COX)–NO system inhibition [40]), it was evident that the application of the NOS blocker L-NAME [40] aggravated the harmful effects of the NSAIDs (i.e., poor anastomosis healing, failed intestine adaptation, and aggravated gastrointestinal, liver, and brain lesions in diclofenac short-bowel rats [40]). BPC 157 completely ameliorated symptoms in all these circumstances, thus, also successfully resolving cyclooxygenase (COX)–NO system inhibition [40].

Notably, we used the distinctive course of the stomach–liver–brain lesions after an overdose of insulin and equal antagonization by BPC 157 therapy to support the more general significance [35]. The characteristics were hypoglycemic seizures, eventually leading to death, which appeared 90 min after insulin, and the severe damage of neurons in the hippocampus and the cerebral cortex. Likewise, the characteristics were no fatal outcome, no hypoglycemic seizures, and markedly fewer damaged brain neurons in BPC 157-treated rats. The particular indications were higher blood glucose levels (glycogen was still present in hepatocytes) [35]. In the prominent calcification of liver blood vessels (both insulin pathways should be inhibited for the calcification [181,182]) in an insulin-administration period of a few hours, BPC 157 administration markedly attenuated [35], the liver appeared to be a normal weight, fatty liver was counteracted, and increased enzyme serum values were counteracted. BPC 157-treated specimens had only occasional small gastric lesions [35]. Thus, we may suggest that BPC 157 recovered both insulin pathways (insulin and glucagon release are under KATP channel control as well as under the control of hypothalamus-brain stem hypoglycemia-induced vagal signaling [181]) during hypoglycemia and regained brain–gut function integrity. The seizures that were markedly attenuated or eliminated [35] might have been, as suggested, the acute brain refueling from the peripheral source and the recovered activity in previously hypoglycemia-silenced brain regions such as the cortex, hippocampus, and amygdala [181].

Thus, these findings might highlight the effective simultaneous recoveries of the brain–gut and gut–brain axes’ functioning with BPC 157 therapy applications. As mentioned before, the advantages of this therapy, including the recovery of the essential bodily system involved, were summarized in the NSAIDs, short-bowel surgery, and insulin brain injury studies [36,37,38,39,40,41,42,43,44]. As a major advantage to verify additional considerable general significance, there was the counteracted leaky gut syndrome in the rats does with indomethacin and the recovery of all of the leaky-gut-syndrome-deranged molecular pathways [41].

Furthermore, the new evidence of the brain-gut axis and gut-brain axis dysfunction or function revealed the harmful significance of the vascular failure to multiorgan failure, peripherally and centrally, and the essential beneficial significance of the rapid activation of the collateral pathways. With this essential vascular effect (i.e., activated azygos vein direct blood flow delivery, upgraded minor vessel to compensate for failed major vessel function), the recovery by BPC 157 therapy functioned to achieve full counteractions of the particular occlusion/occlusion-like syndromes and Virchow triad circumstances [51,52,53,54,56,57,67,80,81,82,83,84]. These occurred with procedures that all largely disabled endothelium function. These was the permanent occlusion of the major vessels, both peripherally and centrally (i.e., inferior caval vein [67,80], hepatic artery, portal vein [54], superior mesenteric artery and/or vein [51,52,53], and superior sagittal sinus [81]). Likewise, this was the application of similar noxious procedures (i.e., absolute alcohol intragastric application [82], lithium overdose [56], isoprenaline-myocardial infarction and reinfarction [57], bile duct permanent occlusion [84], and maintained intra-abdominal hypertension grade III and grade IV [83]). The severe intracranial (superior sagittal sinus) hypertension, portal and caval hypertensions, and aortal hypotension were attenuated/eliminated [51,52,53,56,57,67,80,81,82,83,84]. There were counteractions of the severe lesions in the brain (and intracerebral/intraventricular hemorrhage), lungs (hemorrhage), and severe congestion in the liver, kidney, and gastrointestinal tract. In particular, progressing thrombosis, both peripherally and centrally, and heart arrhythmias and infarctions that would consistently occur were fully counteracted and/or almost annihilated [51,52,53,56,57,67,80,81,82,83,84]. In addition, the otherwise progressing thrombosis, both peripherally and centrally, was fully counteracted by BPC 157 therapy [51,52,53,56,57,80,81,82,83,84]. As a distinctive point of the BPC 157 therapy, i.e. resolving Virchow triad circumstances, whatever the cause, we should point out that there was a large intracerebral and intraventricular hemorrhage fully counteracted along with full counteraction of thrombosis [51,52,53,56,57,80,81,82,83,84] (see also Section 5).

There were shared findings in the central vessels’ occlusion [81] and in the peripheral vessels’ occlusion [51,52,53]. From the superior sagittal sinus occlusion, there was an instant central injury spreading to the periphery (central nervous system to periphery) (i.e., portal and caval hypertensions, aortal hypotension) [81]. Furthermore, as demonstrated and vice versa, from the periphery (periphery to central nervous system), a comparable noxious procedure was initiated at the periphery [51,52,53] and from the periphery (periphery to central nervous system), and intracranial (superior sagittal sinus) hypertension instantly occurred [51,52,53]. As a common outcome, there was multiorgan failure with progressing thrombosis; failed vessels, both centrally and peripherally; congested inferior caval veins; and superior mesenteric veins. The disabled minor vessels, i.e., the azygos vein, might have been unable to compensate for failed vessel function. Consistently, with the reactivating and upgrading of the minor blood vessels, i.e., the azygos vein direct blood flow delivery, the rapid counteraction of the whole syndrome with the therapeutic effects of BPC 157’s application might commonly occur [51,52,53,56,57,67,80,81,82,83,84]. This also might suggest that the brain/heart failure cause–consequence as a whole (given counteracted arrhythmias and thrombosis, peripherally and centrally) might occur in a bidirectional way. Given the commonality between the brain-gut axis and gut-brain axis, and in this BPC 157 significance, there might be an equal therapy effect of the BPC 157 application peripheral, intraperitoneal, intragastric, and central application (i.e. local at the brain tissue) [51,52,53,56,57,67,80,81,82,83,84]. 

Therefore, we might envisage a multitude of lesions and disturbances tightly interconnected in the disabled functioning of the brain–gut axis and gut–brain axis in general [1]. Additionally, we might envisage a large range of therapeutic effects, given the key effect (activation of the collateral pathways) needed to achieve the recovery of the brain lesion that might have been similar to recovery of heart failure and all concomitants similar to multiorgan failure recovery, which are causally inter-related. This included the upgrading of the minor vessels to take and compensate for the failed major vessels, re-establishing the reorganized blood flow. Illustratively, BPC 157 therapy might rapidly upgrade the venous system (azygos vein-direct blood flow delivery), allowing the treated rats to sustain without major injuries even the severe intra-abdominal hypertension, grade III and grade IV, constantly maintained. This included the pushing up of the diaphragm and the most-constrained thoracic cavity for a long period, but this preserved heart function, avoided the rapid transmission of the increased pressure between the three body cavities, markedly attenuated the intracranial (superior sagittal sinus) hypertension, the portal and caval hypertensions were annihilated, and the aortal hypotension was attenuated [84].

Notably, this principle may also be highly acceptable in eye pharmacotherapy as well, such as in the glaucoma model in particular. The cauterization of three of four episcleral veins resulted in rats (only one episcleral vein available, but unable to take full functions) with severely increased intraocular pressure and insurmountable glaucoma presentation [170]. On the contrary, BPC 157 regimens may rapidly normalize intraocular pressure, applied at the early or prolonged post-injury period, and prevent glaucoma or reverse already advanced glaucoma. Therefore, there is rapidly upgrading the remaining episcleral veins to recover all function accordingly [170]. Normalized intraocular pressure in glaucomatous rats [170] corresponded to the counteracted intracranial (superior sagittal sinus) hypertension, portal and caval hypertensions, and aortal hypotension in occlusion/occlusion-like syndromes [51,52,53,56,57,67,80,81,82,83,84], all attenuated/eliminated by BPC 157 therapy. Providing the same and comparable dose ranges and routes of application [51,52,53,56,57,67,80,81,82,83,84], the daily regimen of BPC 157 in glaucomatous rats may be illustrative. It was given locally as drops in each eye (0.4 µg/eye, 0.4 ng/eye; 10 µg/kg, 10 ng/kg) intraperitoneally (last application at 24 h before sacrifice) or per-orally in drinking water (0.16 µg/mL, 0.16 ng/mL, 12 mL/rat until the sacrifice, first application being intragastric) [170].

In conclusion, given the possible significance, the potential of BPC 157 therapy had been effective on all distinctive aspects of the brain lesions thought to be consequent to the failed functioning of the brain–gut axis and brain–gut axis. Likewise, the obtained large range of the therapeutic effects that BPC 157 simultaneously achieved, both peripherally and centrally, might conceptually reestablish the harmony in these axes’ functioning.

These data are summarized in Table 3.

## 5. Thrombosis

There was, commonly noted, marked thrombosis, both centrally and peripherally, during the occlusion/occlusion-like syndrome and progressing brain and internal organs’ lesions. There were intracerebral and interventricular bleedings and hemorrhages in the internal organs as well. Thus, such thromboses (and hemorrhages) might provide particular requirements for the function of the brain–gut and gut–brain axes [51,52,53,56,57,81,82,83,84].

With BPC 157 therapy, the particular beneficial effect on the brain–gut axis and gut–brain function should instantly resolve a considerable number of the failed blood vessels, which might have been unable to be spontaneously activated upon injury in the recruitment of the collateral blood vessels. In resolving the harmful occlusion/occlusion-like syndrome in a way reliant on the given vascular injury, this therapy commonly occurred and might encompassed a large number of distinctive vessel pathways, both peripherally and centrally, as useful collaterals. Specifically, peripherally, there were many vessels involved that were identified. There were veins (the left ovarian [67], inferior mesenteric [54], inferior anterior pancreaticoduodenal, superior anterior pancreaticoduodenal, pyloric [52,53], and azygos [56,57,80,81,82,83,84]) and arteries (the inferior mesenteric artery and inferior anterior pancreaticoduodenal [51]) depending on the injury, with occluded veins (i.e., inferior caval, superior mesenteric) or occluded arteries (superior mesenteric) as alternative rescuing venous or arterial pathways. Centrally, (para)sagittal venous collateral circulation occurred [81]. Thus, the Virchow triad consequences may have been resolved [51,52,53,54,56,57,67,80,81,82]. As evidenced by deep vein thrombosis (inferior caval vein syndrome), consumption thrombocytopenia, given the decreased bleeding, timely carried out to the prolonged bleeding and increased thrombosis, was also counteracted by BPC 157 therapy [67].

Likely, this might reveal the particular combining effect of BPC 157 therapy related to the NO system [43,59,60,69,70,71] and the modulatory interaction between BPC 157 and the NO system as a whole, which is intended to maintain the NO system’s function undisturbed, proved by an equal counteraction of both the NOS blockade (L-NAME) and the counteraction of NO over-activity (L-arginine). As indicative proof, the therapeutic effects of BPC 157 inhibited the pro-thrombotic and hypertensive effects of the NOS blockades (L-NAME) as well as the anti-thrombotic and hypotensive effects of the NOS over-activity (L-arginine) [43,70].

Commonly, this vascular recovery is an immediate therapeutic effect. Occlusion/occlusion-like syndrome presenting as Virchow triad circumstances knock out major veins, which grossly appear disabled (i.e., congested inferior caval and superior mesenteric veins, collapsed azygos vein), meaning the progressing of thrombosis in veins and arteries. Through the therapy, these veins were all quickly recovered and rapidly made fully functional [51,52,53,56,57,81,82,83,84]. The inferior caval and superior mesenteric veins were reversed to normal vein presentations (effect parallel with counteraction of the portal and caval hypertension). Simultaneously, there is the activated azygos direct blood delivery by vein, the heart failure recovery, essential to the instant recovery of thrombosis, peripherally and centrally. Note, this was a general effect, as shown by the assessment of the markedly attenuated (or even eliminated) thrombosis, including a considerable number of vessels, both peripherally and centrally. These were veins (i.e., the inferior caval vein, portal vein, lienal vein, superior mesenteric vein (portal and caval hypertension counteraction), and superior sagittal sinus (counteraction of the intracranial (superior sagittal sinus) hypertension) and arteries (i.e., abdominal aorta, hepatic artery, superior mesenteric artery (counteraction of aortal hypotension)). Thus, the reversal of Virchow triad circumstances is the attenuation or even annihilation of the otherwise progressing hemorrhage (i.e. lung, intracerebral, and interventricular) [51,52,53,56,57,81,82,83,84].

The parallel counteractions of the corresponding lesions [51,52,53,56,57,81,82,83,84] were essential to prove that these effects (i.e., BPC 157 attenuated the spontaneous bleeding [42,43,82,106,183,184] and the prolonged bleeding after anti-coagulants [42,43]; anti-thrombotic agents [42]; and the NOS substrate L-arginine, alone or with amputation (tail, leg) [43] and organ perforation (stomach, cecum) [183,184]), occurred accordingly, while the coagulation pathways were not affected [42,43,44]. The same combined effect occurred with concussive brain injuries [146]. In addition, there was a recovery after the spinal cord compression [147,148]. Furthermore, aggregometry and thromboelastometry studies showed that BPC 157 given with aspirin, clopidogrel, or cilostazol in rats might specifically maintain the function of thrombocytes (i.e., counteracted inhibition on aggregation activated by arachidonic acid, ADP, collagen, and arachidonic acid/PGE1) [44].

Of note, this particular combining effect (counteracted thrombosis, counteracted hemorrhage, both peripherally and centrally) may also be conceptually resolved within the BPC 157 cytoprotection/organoprotection effect (assuming the simultaneous healing of different tissues [1,2,7,85]) combined with wound healing (i.e., implied direct cell protection against direct injury and simultaneous healing [8,73]). There was, in a particular way, resolved wounding (i.e., myotendinous junction recovery, resolved brain and internal organs lesions), as evidenced by the BPC 157 effects in particular [1,2,7,8,73]. Furthermore, wound/cytoprotection terms (i.e., innate endothelium maintenance) might illustrate particularities that might equally resolve both bleeding and thrombosis. Illustrative equations are resolved consequences of the abdominal aorta anastomosis [185] and amputation of the leg or tail [42,43]. Early BPC 157 therapy counteracted the formation of obstructing thrombi. Likewise, the delayed application rapidly annihilated the fully established obstructing thrombi [185], and post-amputation bleeding was decreased [42,43]. Similar equations were in the severe occlusion/occlusion-like syndromes [51,52,53,56,57,67,80,81,82,83,84]. The widespread thrombosis and hemorrhage in the brain and internal organs were both attenuated/eliminated, and both the brain and internal organs lesions were attenuated [51,52,53,56,57,67,80,81,82,83,84]. Thus, this may be the result of the realized healing process after rupturing the blood vessel as a whole and the particular effect on each of all four major events in clot formation and dissolution, fully accomplished in the realization of the healing process. Thus, the demonstrated particular combining effect (counteracted thrombosis, counteracted hemorrhage, both peripherally and centrally) of BPC 157 therapy means the innate distinctive effect that might have been distinctively used depending on the given injury or agent application.

Finally, in addition to the particular maintenance of the thrombocytes’ function [44], an illustrative practical aspect might have been both the prevention of leg disability and rapidly reestablishing leg function [185] that should be mentioned in rats after the creation of abdominal aorta anastomoses [185] in parallel with the counteracting effect on obstructing thrombi [185]. Such a demonstration [185] is in keeping with the findings in the stroke rats, considering counteracted brain lesions and fully recovered function [68]. Thus, there might have been a well-functioning cytoprotection loop, and the translation to the preserved muscle function consistently occurred.

## 6. Conclusions

The brain–gut and the gut–brain axes’ function was perceived through the BPC 157 effects through which it might combine (see, Section 2. Behavior (Table 1), Section 3. Muscle (Table 2), Secion 4. Brain Injury Concomitant Pathology (Table 3), and Section 5. Thrombosis) (for review see, i.e., [1,2,3,4,5,6,7]). The equipotent beneficial therapeutic effects of several routes of BPC 157 application supporting each other’s effect [51,52,53,56,57,67,80,81,82,83,84] may be in keeping with the implied equipotent peripheral and central interplay simultaneously happening. Illustratively, in occlusion/occlusion-like syndromes, and rapidly progressing course, the brain volume proportional to the change in the brain surface area revealed an immediate increase to 120% of the healthy presentation [56,81]. Therefore, with BPC 157 therapy, a rapid decrease in the brain swelling upon local application at the swollen brain implies a prime direct effect, and a rapid decrease in the brain swelling upon intraperitoneal or intragastric administrations verifies a prime systemic effect occurring upon intraperitoneal or intragastric administrations [81]. The effectiveness of both µg and ng regimens means a common therapeutic effect comprising a wide beneficial range of effects, the effects supporting one another [7]. Theoretically, intragastric/per-oral application benefits may verify the BPC 157 concept as an original cytoprotective anti-ulcer peptide (i.e., epithelium, endothelium maintenance, and protection) [7].

In BPC 157 behavioral research, the suggested interactions with the GABA, dopamine, serotonin, and NO systems were based on the evidenced effects of BPC 157 therapy noted in the suited animal models. Therefore, with this general limitation, the anxiolytic, anti-convulsive, and anti-depressant properties were claimed (see, Section 2. Behavior (Table 1)). Likewise, these may be particular interrelations; the obtained effects clearly demonstrated that BPC 157 therapy particularly counteracted the neuroleptic- or L-NAME-induced catalepsy, lithium intoxication, and the schizophrenia-positive (amphetamine; methamphetamine; apomorphine) and schizophrenia-negative (ketamine) symptoms [88,103].

The BPC 157 muscle–brain perception (multimodal muscle axis action function, striated, heart, and smooth muscle, muscle injuries and disturbances, muscle weakness as part of peripheral and central effect) [2,5,6,9] evidently departed from the original brain–gut axis’s perception, explained with gut peptides related mostly to smooth muscle function [10]. Similar to the behavior research (Section 2. Behavior), the BPC 157 muscle–brain perception was based on the evidenced effects of BPC 157 therapy noted in the suited animal models that may have very closely mimicked human circumstances (see, Section 3. Muscle). There were various muscle disturbances in the large range of the periphery–brain system in the BPC 157’s effects; thus, the concept of the multimodal muscles’ axis action function seems fully acknowledged. This acknowledged the impulses passing via the motor cortex–spinal cord-appropriate muscles and impulses passing through various parts of the brain, sending messages back to the motor cortex. This also acknowledged that the general terms of the agent’s effect on muscle disturbances would indicate, in addition to the peripheral (local) effects, the adequate wide range of central effects and the purposive movements (periphery–brain; brain–periphery) [5,9]. Thus, for BPC 157 therapies on muscle–brain perception, we did summarize all these points revealed in the peptide agent’s activity, consistently considered for muscle healing and function in general terms for the realization of the function of the brain–gut axis [5,9]. There were the recoveries of the striated muscle direct lesions; disabilities; systemic disabilities; smooth muscles, sphincter function in particular; vessel function; intestinal adaptation; and heart failure as a whole, along with counteracted arrhythmias and thromboses [2].

Similar to the behavioral research (Section 2. Behavior) and the BPC 157 muscle–brain perception (Section 3. Muscle), the perception of Section 4’s Brain Injury Concomitant Pathology was based on the evidenced beneficial effects of the BPC 157 therapy, verified in the suited animal models evidencing the effective simultaneous recoveries of the brain–gut and the gut–brain axes’ parallel function by BPC 157 therapy, recovering, at the same time, both central and peripheral lesions. These simultaneous recoveries of the central and peripheral lesions (i.e., brain, liver, and gastrointestinal lesions recoveries) were summarized using the application of NSAIDs and/or short-bowel surgery, insulin overdose, leaky gut syndrome, and deranged molecular pathways [36,37,38,39,40,41,42,43,44]. Furthermore, with occlusion/occlusion-like syndromes obtained with permanent major vessel occlusions, both peripherally and centrally, and similar noxious procedures application that all severely disabled endothelium function, the harm of vascular failure to multiorgan failure, both peripherally and centrally, and the essential beneficial significance of the rapid activation of the collateral pathways were summarized. Therefore, there was a considerable extension of the simultaneous recoveries of the central and peripheral lesions (i.e., brain, heart, lung, liver, kidney, and gastrointestinal tract). Simultaneous recoveries also included the simultaneous counteraction of intracranial hypertension (superior sagittal sinus), portal and caval hypertensions, aortal hypotension, ECG disturbances, the counteraction of the progressing thromboses in veins and arteries, both peripherally and centrally, and hemorrhages in the brain and internal organs. As emphasized, recovery by BPC 157 therapy functions to achieve full counteraction against the particular occlusion/occlusion-like syndromes, and Virchow triad circumstances [51,52,53,54,56,57,67,80,81,82,83,84] were attributed to the particular effect on the endothelium function, the rapid upgrading of minor vessels to compensate for failed major vessel function as essential vascular effects, and to activate collateral pathways (i.e., activated azygos vein direct blood flow delivery). Furthermore, there was consistent support for the pleiotropic lesions’ counteraction in the vascular studies [51,52,53,56,57,81,82,83,84]. Furthermore, in previous non-vascular studies, there were particular counteractions of the lesions in the brain [35,36,37,38,40,149,184], lungs [86,186,187,188], liver [35,36,37,38,39,40,49,105,189], kidney [190,191,192], and gastrointestinal tract [35,36,37,38,39,40,41]. In particular, there were counteractions of heart arrhythmias and infarctions [86,114,122,155,156,160,193,194]. Together, these findings might suggest a particular regulatory role.

Commonly, thromboses (and hemorrhages) might require particular requirements from the brain–gut and gut–brain axes’ function and their possible therapies [51,52,53,56,57,67,81,82,83,84] (see, Section 5. Thrombosis). Marked thromboses and hemorrhages centrally and peripherally during the occlusion/occlusion-like syndrome were commonly noted, and the counteraction by BPC 157 [51,52,53,54,56,57,67,80,81,82,83,84] was ascribed also to its significance in wound healing [8,73], realizing the healing process after rupturing the blood vessel as a whole. Therefore, the particular effect on each of all four major events in clot formation and dissolution appeared to be closely involved in the demonstrated particular combining effects (counteracted thrombosis, counteracted hemorrhage, both peripherally and centrally) of BPC 157 therapy [8,73].

This might have all been seen as a network of evidence for the physiologic significance and the revealed BPC 157–vascular system interplay that may have additional physiologic regulatory roles [8,73]. To this point, in situ hybridization and immunostaining studies in humans found BPC 157, as a novel cytoprotection mediator, to be native and stable in human gastric juice, easily applicable, and largely distributed in tissues [8,73].

In addition, outside the original frame of the estimation of brain–gut peptides [10], this review acknowledged lesion recovery as the principle that implied the brain–gut and gut–brain axes to be specifically defined by the range of the effects of the proposed peptide agents [10]. However, a particular regulatory role of the BPC 157 action in the brain–gut axis and the gut–brain axis might also be additionally envisaged. This may be performed based on the large counteracting effect of BPC 157 [5,9] on temperature disturbances (hypothermia (alcohol, reserpine, and serotonin-syndrome) [96,107,120], hyperthermia (yeast, serotonin-syndrome) [107,195]), and convulsions (picrotoxine, strychnine, bicuculline, metrazole [195], insulin [35], paracetamol [36], alcohol withdrawal [96], succinylcholine [158], serotonin syndrome [107], and lidocaine [159]). There may also be a particular effect on pain in seemingly opposite, counteracted local corneal anesthesia (tetracaine oxybuprocaine [160] and intraplantar, axillary, and intratechal (lidocaine, bupivacaine (arrhythmias)) [160,196], counteracted morphine analgesia, and haloperidol potentiation of the morphine analgesia [125] vs. counteracted pain (MgSO_4_ and acetic acid test [195], succinylcholine muscle pain [158], formalin-pain [197], incisional teeth pain [198], and knee pain [199]).

In general, this may be related to reestablishing disabled function and sensitivity as a part of its particular healing effect [8,73]. Illustratively, BPC 157-treated animals with direct muscle [50,150,151,152,153], tendon, ligament, or bone injuries [200,201,202,203,204] quickly started to use the injured area. Intra-articular injection of BPC 157 was very effective for multiple types of knee pain in patients [199].

Based on the similar beneficial effects, similar importance was suggested also for other species (i.e., birds [188] and insects [205,206]). Moreover, BPC 157 had a very safe profile (i.e., without adverse effects in clinical trials (ulcerative colitis, phase II)). The lethal dose (LD1) was not achieved in toxicological studies (for review see [1,2,7,8,73]). A large study conducted by Xu and collaborators with similar results may be taken as confirmation [207]. Finally, the essential finding (i.e., the stable gastric pentadecapeptide BPC 157 therapy rapidly improving the functionality of minor vessels to take over the function of disabled major vessels, reestablishing reorganized blood flow, compensating for failed vessel function) [1] was further elaborated on very recently. Fourier-transform infrared spectroscopy evidenced the rapidly changing lipid contents and protein secondary structure conformations in rat thoracic aorta, being produced instantly by BPC 157 therapy [208], thus emphasizing the, consequently, increased capability of the rat thoracic aorta to function even in the worst circumstances.

Finally, as a disadvantage (or advantage), it should be noted that the concept of cytoprotection/organoprotection, although long ago recognized [13,14,15,16,17,18,19,20,21], was still not brought to fruition. In the meantime, the BPC 157 topic was also approached by some other researchers [209,210,211,212]. Nevertheless, these findings together (for review see [1,2,7,8,73]) may be suggestive of the further application of BPC 157 therapies.

## Figures and Tables

**Table 1:** In BPC 157 behavioral research, the suggested significances and interactions with the GABA, dopamine, serotonin, and NO systems were based on the evidenced effects of the BPC 157 therapy noted in the suited animal models.

References	Effects
[91]	The development of tolerances and physical dependences, are both attenuated.
[93]	Particular anxiolytic effects (i.e., not burying and no more shocks (shock probe/burying), and a greater number of crossing and exploratory rearing behaviors in dark areas (light/dark test)).
[103]	In the counteraction of the negative schizophrenia symptoms in the ketamine-dosed rats, there was an additional anxiolytic effect.
[94]	Antagonization of thiopental-induced general anesthesia (parallel shift of the dose–response curve to the right).
[96,97]	Counteraction of acute and chronic alcohol intoxication.
[91,99]	Counteracted convulsions induced by picrotoxin, isoniazid, and bicuculline.
[115]	In classic antidepressant assays, BPC 157 therapy (Porsolt’s test, chronic stress, reduced duration of immobility) overwhelmed the effect of imipramine.
[107]	Full counteraction of serotonin syndrome as a particular effect.
[108]	Region-specific influences on brain serotonin synthesis in rats in acute and chronic treatments. Serotonin release was increased, particularly related to the innate effect on the substantia nigra structure (alpha-[14C]methyl-L-tryptophan autoradiographic measurements).
[120]	Counteracted disturbances caused by the application of the parkinsongenic neurotoxin 1-methyl-4-phenyl-1,2,3,6-tetrahydrophyridine (MPTP) and vesicle depletion by reserpine application.
[46,70,88,89,121,122,123,124]	Counteracted effects of blockaded dopamine receptors that would appear in haloperidol, fluphenazine, clozapine, and sulpiride applications, peripherally (gastric lesions, sphincter dysfunction, prolonged QTc intervals) and centrally (catalepsy, akinesia).
[125]	Antagonization of the haloperidol potentiation of morphine analgesia. Antagonization of morphine analgesia.
[90,92]	Counteracted disturbances (i.e., stereotypies) in acute and chronic amphetamine applications (i.e., tolerance and reverse tolerance).
[88]	Antagonized disturbances that were characteristic in the courses of amphetamine, methamphetamine, apomorphine, and dopamine (over)-stimulation, in the suited models of the positive-like schizophrenia symptoms.
[103]	Counteraction of the ketamine-negative-like schizophrenia symptoms as NO-related effects: counteraction of cognition dysfunction, social withdrawal, and anhedonia; additional anxiolytic effects exerted (see above).
[103]	Counteraction of the worsening of cognitive dysfunction, anhedonia, and anxiogenic effects induced by L-NAME.
[103]	Counteraction of the worsening of social withdrawal and anxiogenic effects induced by L-arginine.
[90]	Fully avoided striatal dopamine receptor up-regulation and supersensitivity in mice pretreated with haloperidol and, subsequently, challenged with amphetamines. Increased climbing behavior was antagonized.
[88]	Antagonization of L-NAME-induced catalepsy.

**Table 2:** The BPC 157 muscle–brain perception (multimodal muscle axis action function, striated, heart, and smooth muscle, muscle injuries and disturbances, muscle weakness as part of peripheral and central effect) evidently departs from the original brain–gut axis’s perception explained with gut peptides related mostly to smooth muscle function. The BPC 157 muscle–brain perception was based on the evidenced effects of BPC 157 therapy, noted in the suited animal models that may very closely mimic human circumstances.

References	Effects
[50,150,151,152,153,154]	The described improved purposive movement rationale (via the motor cortex–spinal cord-appropriate muscles and vice versa) might conceptualize in the brain–muscle axis function, the healing and function recovery of the myotendinous junction (dissection), the muscle lesion (transection, contusion, and corticosteroid application), and the nerves (transection).
[68]	With counteracted muscle weakness, stroke was counteracted.
[146]	With counteracted muscle weakness, traumatic brain injury was counteracted.
[149]	With counteracted muscle weakness, cuprizone–induced multiple sclerosis-like brain lesions in rats were counteracted.
[147,148]	With counteracted muscle weakness, spinal cord compression lesions were counteracted.
[56,155,156,157]	With counteracted muscle weakness, severe electrolyte disturbances and brain lesions were counteracted.
[96,97,107]	In alcohol intoxication and serotonin syndrome, muscle disturbances were counteracted, along with the antagonization of the whole syndrome.
[158]	Counteraction of the succinylcholine-induced neuromuscular junction blockade.
[45]	Tumor-induced muscle cachexia (i.e., muscle degeneration, inflammation, catabolism, and deranged molecular pathways) was antagonized, and the survival rate increased.
[88,89,90,120]	Catalepsy, akinesia, and tremors with neuroleptic dopamine blockades, NO system blockades, applications of parkinsongenic neurotoxin 1-methyl-4-phenyl-1,2,3,6-tetrahydrophyridine (MPTP), and vesicle depletion by reserpine applications were antagonized.
[51,52,53,54,56,57,67,80,81,82,83,84]	Counteraction of myocardial infarction and myocardial reinfarction, along with brain injury mitigation and severe vascular and multiorgan failure counteraction (activated azygos vein direct blood flow delivery).
[2,51,52,53,54,56,57,67,80,81,82,83,84,86,113,114]	Heart failure was counteracted (including arrhythmias and thrombosis counteracted), in addition to the therapeutic effects of BPC 157 on the various muscle disabilities of a multitude of peripheral and central causes.
[46,48,95,106, 162,163,164,165,166,167,168,169,170,171,172]	Recovery of the distinctive functions of sphincters (lower esophageal sphincter, pyloric sphincter, pupil, urinary sphincter).
[40,174,175]	Intestine recovery after massive intestinal resection, as well-controlled adaptive processes adequately affecting the entire intestinal wall (villus height, crypt depth, and muscle thickness (inner (circular) muscular layer) all accordingly increased) achieved full intestinal anastomosis healing in particular. The counteraction of brain lesions that otherwise might occur regularly after massive bowel resection.
[60]	BPC 157 increased vasorelaxation in the aorta without the endothelium, while BPC 157 modulated the vasomotor tone of an isolated aorta in a concentration- and nitric oxide-dependent manner and induced NO generation, likely by activating the Src-Cav-1-eNOS pathway.

**Table 3:** Brain injury concomitant pathology evidence is based on the noted beneficial effects of BPC 157 therapy, verified in the suited animal models. These models might highlight the effective simultaneous recoveries of the brain–gut and gut–brain axes’ parallel functioning by BPC 157 therapy recovering in both central and peripheral lesions at the same time.

References	Effects
[34,36,37,38,39,40,41,42,43,44]	The counteraction of the noxious course following NSAIDs. These were non-specific NSAIDs, as well as specific NSAIDs. There were simultaneous counteractions of both central and peripheral injuries (brain, liver, and gastrointestinal tract lesions).
[41]	Counteracted leaky gut syndrome in the indomethacin-dosed rats and the recovery of all of the leaky-gut-syndrome-deranged molecular pathways.
[35]	Counteraction of the distinctive course of the stomach–liver–brain lesions after an overdose of insulin (hypoglycemic seizures eventually leading to death, which appeared 90 min after insulin, and severe damage of the neurons in the hippocampus and the cerebral cortex). Prominent calcification in the liver’s blood vessels (both insulin pathways should be inhibited for the calcification) in a few hours of insulin periods was also markedly attenuated.
[51,52,53,54,56,57,67,80,81,82,83,84]	With occlusion/occlusion-like syndromes obtained with permanent major vessel occlusions, both peripherally and centrally, and the application of similar noxious procedures that all severely disabled endothelium functions, the harms of vascular failure to multiorgan failure, both peripherally and centrally, and the essential beneficial significances of the rapid activation of the collateral pathways were summarized. Therefore, there was a considerable extension of the simultaneous recoveries of the central and peripheral lesions (i.e., brain, heart, lung, liver, kidney, and gastrointestinal tract). Simultaneous recoveries also included the simultaneous counteraction of intracranial hypertension (superior sagittal sinus), portal and caval hypertensions, aortal hypotension, ECG disturbances, progressing thrombosis in veins and arteries, peripherally, and hemorrhage in brain and internal organs.

## Data Availability

Data sharing not applicable.
